# Metagenomic analysis of rumen microbial population in dairy heifers fed a high grain diet supplemented with dicarboxylic acids or polyphenols

**DOI:** 10.1186/s12917-016-0653-4

**Published:** 2016-02-19

**Authors:** Roberta De Nardi, Giorgio Marchesini, Shucong Li, Ehsan Khafipour, Kees J. C. Plaizier, Matteo Gianesella, Rebecca Ricci, Igino Andrighetto, Severino Segato

**Affiliations:** Department of Animal Medicine, Production and Health, University of Padova, Legnaro, PD 35020 Italy; Department of Animal Science, University of Manitoba, Winnipeg, MB R3T 2 N2 Canada

**Keywords:** Ruminal acidosis, Rumen microbiota, Fumarate-malate, Polyphenol, Heifers

## Abstract

**Background:**

The aim of this study was to investigate the effects of two feed supplements on rumen bacterial communities of heifers fed a high grain diet. Six Holstein-Friesian heifers received one of the following dietary treatments according to a Latin square design: no supplement (control, C), 60 g/day of fumarate-malate (organic acid, O) and 100 g/day of polyphenol-essential oil (P). Rumen fluid was analyzed to assess the microbial population using Illumina sequencing and quantitative real time PCR.

**Results:**

The P treatment had the highest number of observed species (*P* < 0.10), Chao1 index (*P* < 0.05), abundance based coverage estimated (ACE) (*P* < 0.05), and Fisher’s alpha diversity (*P* < 0.10). The O treatment had intermediate values between C and P treatments with the exception of the Chao1 index. The PCoA with unweighted Unifrac distance showed a separation among dietary treatments (*P* = 0.09), above all between the C and P (*P* = 0.05). The O and P treatments showed a significant increase of the family *Christenenellaceae* and a decline of *Prevotella brevis* compared to C. Additionally, the P treatment enhanced the abundance of many taxa belonging to *Bacteroidetes*, *Firmicutes* and *Tenericutes* phyla due to a potential antimicrobial activity of flavonoids that increased competition among bacteria.

**Conclusions:**

Organic acid and polyphenols significantly modified rumen bacterial populations during high-grain feeding in dairy heifers. In particular the polyphenol treatment increased the richness and diversity of rumen microbiota, which are usually high in conditions of physiological rumen pH and rumen function.

## Background

The rumen is an anaerobic fermentation chamber housing diverse microorganisms such as bacteria, protozoa, fungi and viruses. Bacteria play a key role in rumen fermentation, which in turn greatly impact production and health of dairy cows. [1]. High-grain diets used to improve performance of dairy cows alter microbial communities in the rumen and the symbiosis between the host and these communities by causing the production of excessive amounts of organic acids (volatile organic acid (VFA) and lactate) [2–4]. High concentrations of VFA and lactate can exceed the buffering capacity of the rumen resulting in major changes in the rumen environment, such as a pH depression, and reduction in the populations of many beneficial bacteria [1, 5, 6]. Large individual variability of the rumen bacterial community, even in animals feeding on the same ration have been reported. [7, 8]. Other studies [1, 6, 7] showed that dietary changes, like an increased inclusion of cereals in the ration, lead to shifts in the microbial community even though the ruminal microbiota was able to maintain a stable core of bacterial taxa. The populations of starch-fermenting and lactic acid-utilizing bacteria increase when high starch diets are fed [9]. An excessive depression in rumen pH causes a reduction in the number of cellulolytic bacteria [2], major shifts in bacterial populations, and increases the lysis of Gram-negative bacteria resulting in a higher concentration of free lipopolysaccharide (LPS) endotoxins in rumen digesta [2, 10, 11]. When the translocation of free LPS from the rumen and/or lower gut into the interior circulation occurs, an immune response in the host is induced, causing metabolic alterations. Moreover, the increase in proportion of bacteria such as *Enterobacteriacae* during high starch feeding heightens the presence of virulence factors (fimbrial adhesins, heat-stable and heat-labile toxins and inflammatory peptides), which have the potential to cause inflammation [2, 10, 12]. Nutritional strategies to prevent the onset of SARA and translocation of LPS in dairy cows are based on feeding sufficient amounts of physically effective fiber (peNDF) and modulating the amount of easily degradable starch in the diet [13]. In addition the use of feed supplements to enhance the rumen microbial community and subsequently ruminal fermentation has also been suggested for this purpose. These supplements include the use of yeasts, like *Saccharomyces cerevisiae*, strain CNCM I-1077 [14], probiotic bacteria, like *Megasphaera elsdenii*, strain H6F32 [15], and dicarboxylic acids [16], flavonoids [17] and essential oils [18]. It has been suggested that the dicarboxylic acids malate and fumarate attenuate the ruminal pH drop during high grain feeding [16], and that these acids increase the activity of the succinate-propionate metabolic pathway in several rumen bacteria, resulting in increased lactic acid uptake and production of propionate [19, 20]. In *in vitro* rumen fermentative experiments, several essential oils (EO), or blends of EO have been demonstrated to enhance rumen fermentation [18, 21]. However, few *in vivo* studies have investigated the effects of EO on rumen fermentation and bacterial populations [22]. Furthermore, the effects of addition of polyphenolic compounds like flavonoids to diets of dairy cows may include prevention of the pH reduction and the decrease of the acetate-to-propionate ratio due to an increase of the numbers of lactate-consuming and propionate-producing bacteria [17].

A previous study described the effect of organic acid (O) and polyphenols (P) on reticular pH drop and acute phase response in dairy heifers fed a high grain diet [23]. In this manuscript we report the effects of these supplements on rumen bacterial populations in dairy heifers fed a high-grain diet.

## Results

Illumina sequencing produced 317,369 sequences across treatments. The average numbers of sequences were 16,413, 18,814 and 28,247 for the C, O and P treatment, respectively. The number of the generated sequences was not affected by treatment (*P* = 0.60).

The P treatment led to the highest richness of the rumen microbial population (Table 1), characterized by the highest number of observed species (*P* < 0.10) and the highest value of ACE (*P* < 0.05). The O treatment resuted in intermediate values between P and C for both the number of species observed and the index ACE.

**Table 1:** Number of observed species, richness (Chao1 and ACE) and diversity estimators (Shannon, Simpson and Fisher’alfa) in control (C), organic acid (O) and polyphenols (P) dietary treatments

	Observed species	Richness		Diversity		
		Chao1	ACE	Shannon	Simpson^1^	Fisher’s alpha
Control	2,116^β^	3,420^b^	3,731^b^	5.9	0.99	645^β^
Organic acid	2,255^α, β^	3,834^b^	4,214^a, b^	6.1	0.99	691^α, β^
Polyphenols	2,742^α^	7,164^a^	6,927^a^	6.1	0.98	890^α^
SEM	188.7	653.1	711.8	0.22	(0.99–0.98)	78.2
*P*-value	0.08	0.03	0.06	0.90	0.93	0.07

^1^Statistical analysis was conducted on natural logarithm (ln) transformed data that are presented as ln back transformed and 95 %-confidence interval in brackets

^a, b^Within column indicate statistical differences (*P* < 0.05); ^α, β^Within column indicate statistical differences (*P* < 0.10)

The Chao1 index was higher during the P treatment (*P* < 0.05) compared to the C and O treatments. Moreover, Fisher’s alpha diversity tended (*P* < 0.10) to be higher during the P treatment compared to the C treatment, whereas the O resulted in intermediate values. The Shannon and Simpson indices were not affected by treatments.

**Fig. 1:**
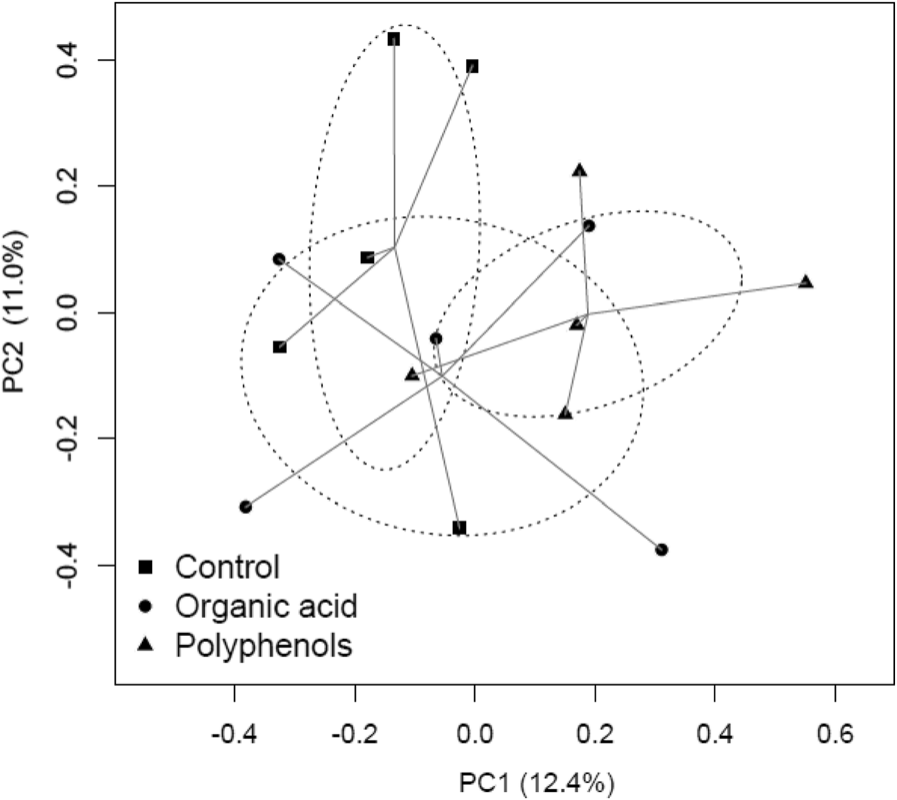
Principal coordinate analysis (PCoA) using unweighted Unifrac to explore dissimilarities in microbial composition among dietary treatments (Control, C; Organic acids, O and Polyphenols, P). The axes (PC1 = 12.4 % and PC2 = 11.0 %) account for 23.4 % of the total variation of the model

The PERMANOVA analysis showed a significant (*P* < 0.10) effect of treatments on Bray-Curtis and unweighted Unifrac distances (Table 2). In addition, differences were observed in bacterial population among periods (*P* = 0.02 and *P* = 0.06, respectively). However the interaction of treatment and period (D × P) on these measures was not significant pointing out the lack of a carry-over effects of treatments. The multiple-comparisons based on Bray-Curtis distance showed only a tendency (*P* = 0.11) towards a significant difference between dietary treatments (C vs P), while the multivariate analysis based on unweighted Unifrac distance indicated a significant (*P* = 0.05) separation between the C and P treatments (Table 2). The comparison of the bacterial communities by principal coordinate analysis (PCoA) with unweighted Unifrac distance (Fig. 1) confirmed a separation between the C and P treatments.

**Table 2:** PERMANOVA analysis of the effect of dietary treatments, periods and heifers on rumen bacteria dissimilarities based on Bray-Curtis and unweighted Unifrac

Source	Bray-curtis		Unweighted unifrac	
	pseudo-*F*	*P*-value	pseudo-*F*	*P*-value
Dietary treatment	1.208	0.10	1.155	0.09
Period	1.328	0.02	1.174	0.06
Heifer	1.175	0.11	1.135	0.09
*Pair-wise tests*				
Control vs Organic acid		0.14		0.22
Organic acid vs Polyphenols		0.30		0.15
Control vs Polyphenols		0.11		0.05

*P*-values were calculated on 999 possible permutations

The abundance of the 20 phyla detected by using Illumina was not affected by dietary treatment. *Bacteroidetes*, *Firmicutes, Tenericutes* and *Euryarchaeota* bacterial phyla were abundant (>1 %) and accounted for 94.8 % of the total bacterial community. *Spirochaetes*, *Proteobacteria*, *Actinobacteria*, *Fibrobacteres*, *Chloroflexi*, *Cyanobacteria* and *Verrucomicrobia* were in low abundance (0.1–1 %), and unclassified bacteria accounted for 2.3 % of the total bacterial community.

**Fig. 2:**
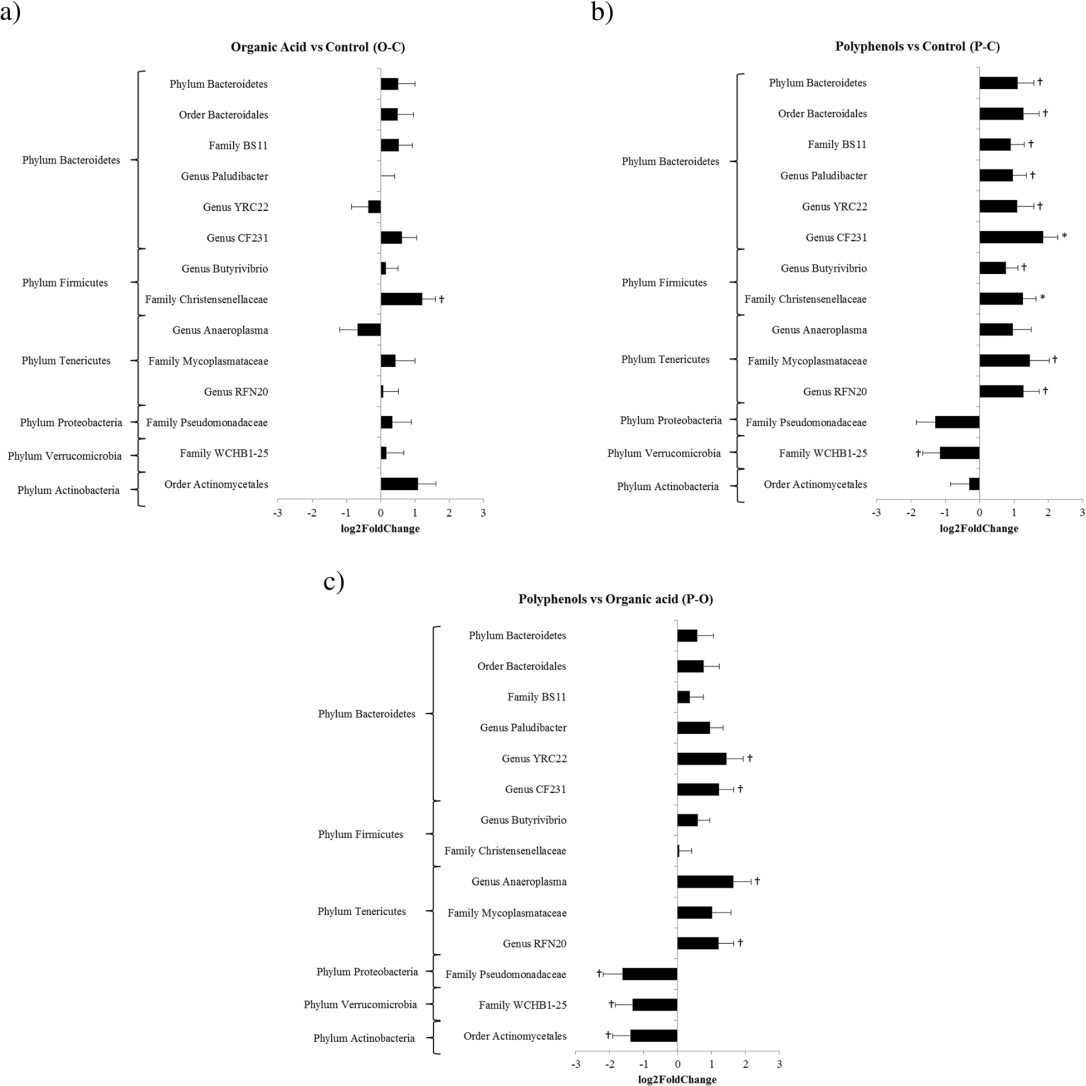
**a**, **b**, **c**. Relative changes (log_2_ fold) in rumen bacterial taxa (based on the classification of 16s RNA gene) in the comparison of **a** organic acid vs control, **b** polyphenols vs control, and **c** polyphenols vs organic acid determinated with Illumina. †: *P* < 0.10; *: *P* < 0.05. Bar = standard error

Illumina sequencing detected 32 classes, 55 orders, 87 families and 88 genera. The predominant sequences (>1 %) within *Bacteroidetes* belong to order *Bacteroidales* (22.7 %), genus *Prevotella* (21.2 %), genus *Paludibacter* (2.24 %), family *BS11* (2.04 %), family *S24*-*7* (1.77 %), and genus *CF231* (1.06 %). The predominant sequences in *Firmicutes* were genus *Butyrivibrio* (4.30 %), and family *Christensenellaceae* (2.43 %), and in phylum *Tenericutes* they were order *RF39* (1.74 %). In particular, the cellulolytic bacteria *Ruminococcus*, *Treponema* and *Fibrobacter* represented 8.15, 0.58 and 0.19 %, respectively on average of total bacteria. The relative abundance of the starch-fermenting bacteria were low and included: *Bifidobacterium* (0.43 %), *Eubacterium* (0.03 %), *Selenomonas* (0.02 %), *Succinivibrio* (0.02 %), and *Ruminobacter* (<0.01 %). The relative abundance of *Butyrivibrio* (4.30 %) was high, while *Succimonas*, *Lactobacillus* and *Streptococcus* were not detected. Among lactic acid-utilizing bacteria, *Anaerovibrio* (0.01 %) was detected, whilst *Fusobacterium* was not detected. The statistical analyses of these data (OTUs–Illumina), refering to the single taxa, showed a total of 19 significant pair-wise comparisons (O vs C, P vs C, and P vs O) as reported in Fig. 2. The comparison between O and C showed that only the family *Christensenellaceae* was significantly (*P* < 0.05) more abundant in the O treatment (Fig. 2a). The greatest number of significant differences between OTUs (11) were observed in the comparison between the P and C treatments. In particular, the P dietary treatment showed a significant higher number of OTUs in the case of phylum *Bacteroidetes*, order *Bacteroidales* (phylum *Bacteroidetes*), family BS11 (phylum *Bacteroidetes*), genus *Paludibacter* (phylum *Bacteroidetes*), genus YRC22 (phylum *Bacteroidetes*), genus CF231 (phylum *Bacteroidetes*), genus *Butyrivibrio* (phylum *Firmicutes*), family *Christensenellaceae* (phylum *Firmicutes*), family *Mycoplasmataceae* (phylum *Tenericutes*), and genus RFN20 (phylum *Tenericutes*). The opposite occurred for the family WCHB1-25 (phylum *Verrucomicrobia*) (Fig. 2b). Moreover, during the P treatment the abundance of genera YRC22 (phylum *Bacteroidetes*), CF231 (phylum *Bacteroidetes*), *Anaeroplasma* (phylum *Tenericutes*), and RFN20 (phylum *Tenericutes*), was increased compared to the O treatment; the contrary for the families *Pseudomonadaceae* (phylum *Proteobacteria*), WCHB1-25 (phylum *Verrucomicrobia*) and the order *Actinomycetales* (phylum *Actinobacteri*a) (Fig. 2c).

**Fig. 3:**
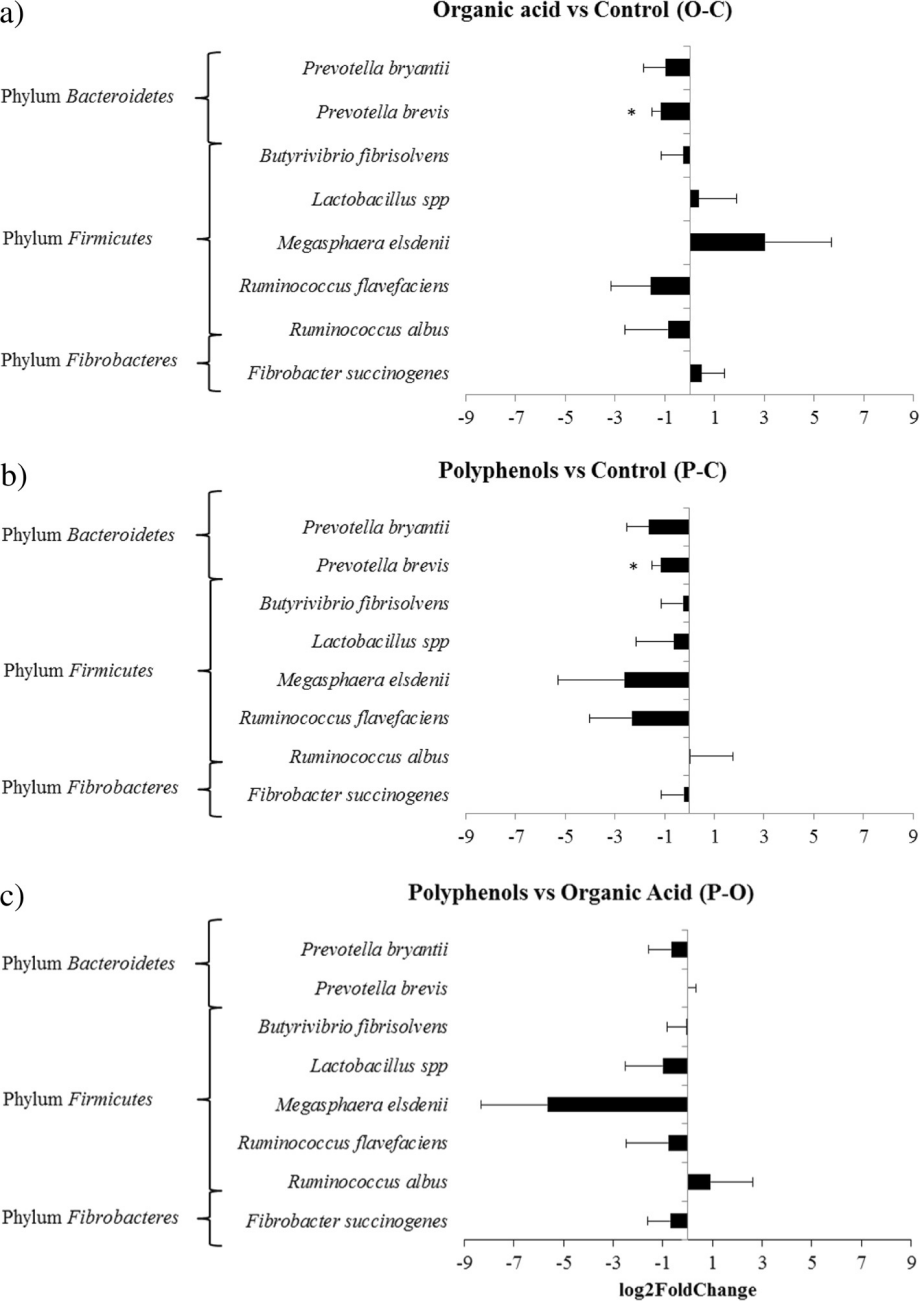
Relative changes (log2 fold) in predominant rumen microorganisms in the comparison of **a** organic acid vs control, **b** polyphenols vs control, and **c** polyphenols vs organic acid determined with quantitative real-time PCR. *: *P* < 0.05. Bar = standard error

The comparison of single species based on qPCR and submitted to the linear MIXED model showed a significant effect of the treatments only in the case of *Prevotella brevis.* This species was more abundant (*P* = 0.03) during the C treatment compared to the other treatments (Fig. 3).

## Discussion

This study was conducted to investigate the effects of the two feed supplements, i.e. organic dicarboxylic acids (O) and polyphenols (P), on the rumen microbiota in dairy heifers fed a high-grain diet. The effects of these additives on reticular pH, VFA, lactate and immune response was published earlier by De Nardi et al. [23]. In this companion study both additives, O and P, attenuated the reticular pH drop due to high-grain feeding wich resulted in 16, 18 and 199 min/d spent below pH 5.6, for O, P and control, respectively. In particular, the P treatment was more effective in reducing the inflammatory response that resulted from the high grain feeding without interfering with dry matter intake (DMI), whereas the O treatment caused the highest decline of the acetate to propionate ratio.

Both supplements enhanced the richness and α-diversity of the rumen microbiota compared to control. This is in agreement with other studies [1, 5, 22], which report a higher richness and diversity for cows with physiological rumen pH and rumen function compared to cows with impaired rumen parameters. This finding could have been related to the different ruminal pH conditions found in heifers fed P and O treatments, which spent daily less time below pH 5.6 [23] compared to heifers on the control treatment. However, the richness and diversity were higher in the P than in the O treatment, despite the similar rumen pH values of these treatments, suggesting that these changes are also based on other factors than ruminal pH. Other authors [7] in fact found that fermentation products, like VFA, have a large influence on the composition of the bacterial community in the rumen. Differences in the composition of the rumen microbiota between the O and P treatments could be explained by the different mechanisms of action of the supplements. However because of the complexity of the interactions among taxa it is difficult to clearly understand these biochemical processes in detail. The O treatment may have increased electron sinks for H_2_ allowing an increase of lactate utilization by bacteria which use the succinate-propionate pathway to synthesize succinate and (or) propionate [16, 19]. This hypothesis was confirmed by the lowest acetate to propionate ratio observed during the O treatment as reported by De Nardi et al. [23]. The blend supplemented during the P treatment contained flavonoids, e.g. polyphenols. Thanks to antimicrobial activity of theser products [24], the P treatment was reported to promote bacteria that metabolize lactate to VFA avoiding lactate accumulation in the rumen [17]. The higher richness and α-diversity found during the P treatment, which represent the diversity within sample [25], could be the consequence of a restraining effect towards some of the dominant taxa present in the rumen during high grain feeding, thereby leaving nutrient substrates available for other microbial groups. Despite differences in richness and diversity among treatments, the multivariate analysis based on PCoA explained a small percentage of the variance. Based on the microbial analysis results, the PCoA discriminant model was able to separate the control treatment from the P and O treatments. This discrimination demonstrated an association between the rumen pH drop resulting from high grain feeding and a decrease of *Prevotella brevis* as reported by Fernando [1], and an increase of abundance of the family *Christensenellaceae*. To the best of our knowledge this is the first contribute that reported a relation between rumen pH changes and the abundance of family *Christensenellaceae*. The change in the abundance of this family could be used as a microbial marker for a balanced ruminal pH condition. This relationship needs to be confirmed by further studies.

Results from Illumina sequencing showed that treatments affected the abundance of various phyla, orders, families, and genera. Among the species investigated with qPCR only *Prevotella brevis* was reduced by the P and O treatments. Matsui et al. and Khafipour et al. [5, 26] reported that *Prevotella spp.* are involved in starch degradation and utilization and thus grow better at low pH conditions.

Among the OTUs detected by Illumina sequencing, the family *Pseudomonadaceae*, order *Actinomycetales,* and *Verrucomicrobia* were reduced by the P treatment compared to the O treatment. These reductions were probably due to a potential antimicrobial activity of flavonoids as reported earlier [17, 24]. Decreasing the presence of these microbials was associated with the increase of several genera belonging to *Bacteroidetes* and *Tenericutes*. Similar trends were observed when comparing the P treatment with the control treatment, but in this case there were also increases in the abundances of some *Firmicutes,* such as genus *Butyrivibrio*. The high grain diet fed to heifers in this study resulted in a rumen microbiota typical of high concentrate feeding [6], although especially the P treatment affected this microbiota. In fact, the higher pH and the antimicrobial activity towards some microbial families, due to the P treatment, allowed an increase of bacteria which are usually favoured by low starch diets and a higher reticular pH. These include bacteria belonging to the family BS11 as reported by Zened et al. [22] and *Paludibacter* [27]. The latter was found to decrease when steers were transitioned from a hay-based to cereal grain-based diet. Even the genus *Butyrivibrio* showed the same response, i.e. increasing due to a higher rumen pH, since it is usually involved in fibre degradation [28].

The use of supplements over short times altered microbial populations. This shows that the rumen is a highly dynamic environment, and that the microbiota are capable of adapting to dietary changes. Polyphenols, in particular, were found to increase the richness and diversity of rumen microbiota in heifers feeding high starch diets.

## Conclusions

Both additives (organic acid and polyphenols) changed microbial communities in the rumen, including the decline of *Prevotella brevis* and the increase of *Christensenellaceae*. These modifications were partially explained by the attenuation of the low reticulo-rumen pH caused by both additives. Polyphenols increased the microbial biodiversity in terms of richness and α-diversity more than organic acids, suggesting a benefit in using this additive in cattle fed high-grain diets. Furthermore, principal coordinate analysis demonstrated the discrimination between control and supplemented diets. The differences in the composition of the microbiota in the rumen between organic acid and polyphenol supplemented cows were likely due to an antimicrobial activity of the latter that widened the competition among different taxa.

## Methods

### Animals, experimental design and dietary treatments

The experimental protocol and all the procedures used in this study were approved by the Animal Ethics Committee of the University of Padova, Italy (CEASA, approval number 73/2012).

The experimental design and dietary treatments are described in De Nardi et al. [23]. Briefly, the study was carried out using six purebred Italian Holstein-Friesian non- pregnant heifers with an average body weight (BW) of 556 ± 33 kg (mean ± SD) according to a 3 × 3 Latin square design, with 3 experimental periods and 3 dietary treatments.

During each experimental period that lasted 22 days, a low starch (LS) diet was given *ad libitum* to the heifers for 14 d, followed by feeding a high starch (HS) diet for 8 d (from d 15 to d 22). Diets (Table 3) were provided as total mixed ration (TMR) once daily at 0800 h. From the d 18 to the d 22, barley meal was top dressed and its dose was gradually increased from 0.5 to 1.5 kg (250 g/day) to cause a rumen pH drop. Diet composition and the protocol to reduce rumen pH were similar to those used in an earlier study [29] and were adopted to prevent acute ruminal acidosis. During the HS diet feeding, the heifers were offered one of the following three dietary treatments: i) no supplement, C treatment; ii) a daily dose of 60 g of fumarate-malate mixture (RumenStabiliser®, DR. Eckel, Niederzissen, Germany), O treatment; iii) a daily dose of 100 g polyphenol-essential oil mixture (Anta®Phyt RU, DR. Eckel, Niederzissen, Germany), P treatment. The O supplement is a buffer and an organic acid mixture made of magnesium fumarate, sodium acetate, malic acid and sodium bicarbonate, whereas P is a mixture made of plant extracts, characterised by a high content of phenolic compounds comprising mostly flavonoids (1.88 mg/g). Feed samples were dried at 60 °C for 48 h, ground to 1-mm and then analysed for DM, crude protein (CP), crude fat and crude ash according to AOAC [30]. The NDF and ADF were analyzed according to Van Soest et al. [31], whilst the starch content was determined using high performance liquid chromatography [32].

**Table 3:** Ingredients and proximate composition of diets fed to heifers

	Diet^a^	
Item	LS	HS
Ingredients, % of DM		
Corn meal (0.5 mm)	23.0	34.0
Hay	24.0	22.0
Dehydrated alfalfa hay	18.0	16.0
Extruded soybean hull	7.0	2.0
Barley meal	7.0	9.0
Straw	6.5	5.5
Molasses	2.0	2.0
Sugar beet dry pulps	4.0	1.0
Corn gluten meal	6.0	2.0
Sunflower	-	2.0
Soybean meal	-	2.0
Vitamin and mineral mix	1.5	1.5
Extruded de-hulled soybean	1.0	1.0
Proximate composition		
DM, %	88.9	88.8
Crude protein, % of DM	12.5	12.5
Crude fat, % of DM	3.5	3.3
NDF, % of DM	39.8	33.6
Crude ash, % of DM	7.8	7.4
ADF, % of DM	21.4	19.2
NFC,^b^ % of DM	36.4	43.2
Starch, % of DM	24.0	30.0

^a^Diets: *LS* low starch, *HS* high starch

^b^NFC = 100 – (NDF + crude protein + crude fat + crude ash)

Sampling and analyses of ruminal fluid were also reported by De Nardi et al. [23]. Rumen fluid was transferred to 50-ml sterile tubes and immediately frozen in liquid nitrogen and then stored at −80 °C until analysis for quantitative real-time PCR (qPCR) and Illumina sequencing.

### DNA extraction

Frozen rumen fluid samples were thawed at room temperature quickly and then kept on ice. One millilitre of rumen fluid was centrifuged at 15,000 × *g* followed by removing the supernatant. The DNA was extracted from pellets (200 mg of each sample) using ZR fecal DNA MiniPrep^TM^ kit (Zymo Research Corp., Irvine, CA), which included a bead-beating step for the mechanical lysis of the microbial cells.

At the last step of the procedure, DNA was eluted from the column with elution buffer, and the concentration and purity of DNA were subsequently determined using a A260/280 spectrophotometer (NanoDrop 2000, Thermo Scientific, DE, USA). The DNA in all samples was diluted with the same elution buffer to a final nominal concentration of 20 ng/μl and quality was checked by PCR amplification of the 16S rRNA gene using universal primers 27 F (5’-GAAGAGTTTGATCATGGCTCAG-3′) and 342R (5′-CTGCTGCCTCCCGTAG-3′), as described by Khafipour et al. [5]. Amplicons were verified by agarose gel electrophoresis [5].

The DNA samples for qPCR analysis were diluted 2 ng/μl and aliquoted into 10 μL/vials, which is sufficient for testing one set of primers, in order to avoid repeated freeze-thaw cycles. All DNA samples were stored at −80 °C until analysis.

### Sequencing

Library Construction and Illumina Sequencing.

Throughout the use of modified F515/R806 primers [33], the V4 region of 16S rRNA gene was adopted for PCR amplification. The reverse PCR primer was indexed with 12-base Golay barcodes and samples were analysed in the PCR reaction. ZR-96 DNA Clean-up Kit™ (ZYMO Research, CA, USA) was used to purify the PCR products. The V4 library was quantified using Picogreen dsDNA (Invitrogen, NY, USA). Multiple dilution steps with pre-chilled hybridization buffer (HT1) (Illumina, CA, USA) were used to obtain a 5 pM concentration of the pooled amplicons, which was determined using Qubit® 2.0 Fluorometer (Life technologies, ON, Canada). In order to reduce unbalanced and biased base compositions, 15 % of PhiX control library was spiked into the amplicon pool. Customized sequencing primers for read1 (5′-TATGGTAATTGTGTGCCAGCMGCCGCGGTAA-3′), read2 (5′-AGTCAGTCAGCCGGACTACHVGGGTWTCTAAT-3′) and index read (5′-ATTAGAWACCCBDGTAGTCCGGCTGACTGACT-3′) were synthesized and purified using polyacrylamide gel electrophoresis (Integrated DNA Technologies, IA, USA). A MiSeq platform (Illumina, CA, USA) was used to perform the 150 paired-end sequencing reaction.

### Quantitative PCR analysis

Rumen fluid samples were analyzed by qPCR using primers for *Eubacteria*, *Prevotella brevis*, *Prevotella bryantii*, *Fibrobacter succinogenes*, *Megasphaera elsdenii*, *Ruminococcus albus*, *Ruminococcus flavefaciens*, *Butyrivibrio fibrisolvens*, *Lactobacillus spp*., and *Streptococcus bovis* as described in Wang et al. [34], Ozutsumi et al. [35], Denman and McSweeney [36], Khafipour et al. [5], Fernando et al. [1]. Real-time PCR was carried out using a CFX connect Real Time system (Bio-Rad Laboratories, Inc., USA). Each reaction mixture was run in triplicate in a volume of 15 μl in optical reaction plates (Thermo Fisher Scientific, U.K.) sealed with optical adhesive film (Applied Biosystems, Foster City, CA, USA). Amplification reactions were carried out with 7.5 μl Power SYBR green PCR master mix (Applied Biosystems, Foster City, CA, USA) mixed with the selected primer set at a concentration of 0.5 μM for each primer and 10 ng of genomic DNA. Amplification consisted of one cycle of 95 °C (10 min), 40 cycles of denaturation at 95 °C (15 s), and annealing/extension at 60 °C (1 min). Final melting analysis was conducted by slow heating from 65 °C to 95 °C in order to assess the specificity of the primer set. Data were normalized for *Eubacteria* using the universal bacteria 16S RNA gene primer sets, which detect all bacterial strains.

### Bioinformatic analysis

The PANDAseq assembler [37] was used to merge overlapping paired-end Illumina fastq files. All the sequences with mismatches or ambiguous calls in the overlapping region were removed. The QIIME software [38] was used to analyze the output fastq file and the reads obtained were filtered and selected to discard the unsuitable ones. The sequences were assigned to Operational Taxonomic Units (OTUs) after being processed by UCHIME [39] and UCLUST [40]. The RDP classifier [41] was used to find correspondence between each OTU and known taxa. Chao1, abundance based coverage estimated (ACE), Shannon and Simpson indices were calculated using the Phyloseq package (version 1.9.2) in R (version 3.0.2).

### Statistical analyses

After verifying the normality of residuals (PROC UNIVARIATE) of the observed species, richness, diversity and qPCR microbial variables were analyzed using the MIXED procedure with a compound symmetry structure using a linear model that included the fixed effects dietary treatment, period and their interaction, and the random effect of animal. If significant treatment effects were detected (*P* < 0.10), the LS*means* were compared using the probability of differences (PDIFF) option and the Tukey adjustment test. In order to obtain a normal distribution and homogeneous residual error, Simpson (diversity) and qPCR microbial variables were log transformed (SAS, release 9.3, 2010). Using the same linear model, the β-diversity indices (Bray-Curtis and unweighted Unifrac) were submitted to distance-based permutational multivariate analyses of variance (PERMANOVA) according to Anderson [42].

Principal coordinate analysis (PCoA) was conducted to evaluate differences in community structure among dietary treatments (β-diversity). PCoA was generated using unweighted Unifrac distance [43] (R package vegan version 2.0–10, 2013).

At the bacterial taxa level, pair-wise differential expression analyses were performed in the nbinomWaldTest method of DESeq2 [44] according to a statistical model consisting in the effects of dietary treatment, period and heifer; these were considered significant at *P* < 0.05 and trends were discussed at 0.05 < *P* < 0.10 (DESeq2, R package version 1.5.34, 2012).

## Abbreviations

ACE: abundance based coverage estimated
ADF: acid detergent fiber
BW: body weight
C: control
CP: crude protein
DM: dry matter
DMI: dry matter intake
EO: essential oils
HS: high starch
LS: low starch
NDF: neutral detergent fiber
O: organic acid
OTU: operational taxonomic unit
P: polyphenols
PCoA: principal coordinate analysis
PDIFF: probability of differences
qPCR: quantitative real-time polymerase chain reaction
TMR: total mixed ration

## References

[1] Fernando SC, Purvis HT, Najar FZ, Sukharnikov LO, Krehbiel CR, Nagaraja TG (2010). Rumen microbial population dynamics during adaptation to a high-grain diet. Appl Environ Microbiol.

[2] Plaizier JC, Krause DO, Gozho GN, McBride BW (2009). Subacute ruminal acidosis in dairy cows: The physiological causes, incidence and consequences. Vet J.

[3] Zebeli Q, Metzler-Zebeli BU (2012). Interplay between rumen digestive disorders and diet-induced inflammation in dairy cattle. Res Vet Sci.

[4] Marchesini G, De Nardi R, Gianesella M, Stefani AL, Morgante M, Barberio A (2013). Effect of induced ruminal acidosis on blood variables in heifers. BMC Vet Res.

[5] Khafipour E, Li S, Plaizier JC, Krause DO (2009). Rumen microbiome composition determined using two nutritional models of subacute ruminal acidosis. Appl Environ Microbiol.

[6] Hook SE, Steele MA, Northwood KS, Dijkstra J, France J, Wright ADG, McBride BW. Impact of subacute ruminal acidosis (SARA) adaptation and recovery on the density and diversity of bacteria in the rumen of dairy cows. FEMS Microbiol Ecol. 2011;78(2):275–84. 10.1111/j.1574-6941.2011.01154.x 21692816

[7] Golder HM, Denman SE, McSweeney C, Wales WJ, Auldist MJ, Wright MM, Marett LC, Greenwood JS, Hannah MC, Celi P, Bramley E, Lean IJ. Effects of partial mixed rations and supplement amounts on milk production and composition, ruminal fermentation, bacterial communities, and ruminal acidosis. J Dairy Sci. 2014;97:5763–85. 10.3168/jds.2014-8049 24997657

[8] Jami E, Mizrahi I (2012). Composition and Similarity of Bovine Rumen Microbiota across Individual Animals. PLoS One.

[9] Nagaraja TG, Titgemeyer EC (2007). Ruminal acidosis in beef cattle: the current microbiological and nutritional outlook. J Dairy Sci.

[10] Plaizier JC, Khafipour E, Li S, Gozho GN, Krause DO (2012). Subacute ruminal acidosis (SARA), endotoxins and health consequences. Anim Feed Sci Technol.

[11] Mao SY, Zhang RY, Wang DS, Zhu WY (2013). Impact of subacute ruminal acidosis (SARA) adaptation on rumen microbiota in dairy cattle using pyrosequencing. Anaerobe.

[12] Khafipour E, Plaizier JC, Aikman PC, Krause DO (2011). Population structure of rumen Escherichia coli associated with subacute ruminal acidosis (SARA) in dairy cattle. J Dairy Sci.

[13] Zebeli Q, Aschenbach JR, Tafaj M, Boguhn J, Ametaj BN, Drochner W (2012). Invited review: Role of physically effective fiber and estimation of dietary fiber adequacy in high-producing dairy cattle. J Dairy Sci.

[14] Bach A, Iglesias C, Devant M (2007). Daily rumen pH pattern of loose-housed dairy cattle as affected by feeding pattern and live yeast supplementation. Anim Feed Sci Technol.

[15] Long M, Feng WJ, Li P, Zhang Y, He RX, Yu LH (2014). Effects of the acid-tolerant engineered bacterial strain *Megasphaera elsdenii* H6F32 on ruminal pH and the lactic acid concentration of simulated rumen acidosis in vitro. Res Vet Sci.

[16] Nisbet DJ, Callaway TR, Edrington TS, Anderson RC, Krueger N (2009). Effects of the dicarboxylic acids malate and fumarate on E. coli O157:H7 and Salmonella enterica typhimurium populations in pure culture and in mixed ruminal microorganism fermentations. Curr Microbiol.

[17] Balcells J, Aris A, Serrano A, Seradj AR, Crespo J, Devant M (2012). Effects of an extract of plant flavonoids (Bioflavex) on rumen fermentation and performance in heifers fed high-concentrate diets. J Anim Sci.

[18] Calsamiglia S, Busquet M, Cardozo PW, Castillejos L, Ferret A (2007). Invited review: Essential oils as modifiers of rumen microbial fermentation. J Dairy Sci.

[19] Martin SA (1998). Manipulation of ruminal fermentation with organic acids: a review. J Anim Sci.

[20] Khafipour E, Krause DO, Plaizier JC (2009). A grain-based subacute ruminal acidosis challenge causes translocation of lipopolysaccharide and triggers inflammation. J Dairy Sci.

[21] Marchesini G, De Nardi R, Ricci R, Andrighetto I, Serva L, Segato S (2014). Effects of carbohydrase inhibiting compounds on in vitro rumen fermentation. Ital J Anim Sci.

[22] Zened A, Combes S, Cauquil L, Mariette J, Klopp C, Bouchez O (2013). Microbial ecology of the rumen evaluated by 454 GS FLX pyrosequencing is affected by starch and oil supplementation of diets. FEMS Microbiol Ecol.

[23] De Nardi R, Marchesini G, Plaizier JC, Li S, Khafipour E, Ricci R (2014). Use of dicarboxylic acids and polyphenols to attenuate reticular pH drop and acute phase response in dairy heifers fed a high grain diet. BMC Vet Res.

[24] Lopez-Lazaro M (2009). Distribution and biological activities of the flavonoid luteolin. Mini Rev Med Chem.

[25] McCann JC, Wickersham TA, Loor JJ (2014). High-throughput methods redefine the rumen microbiome and its relationship with nutrition and metabolism. Bioinform Biol Insights.

[26] Matsui H, Ogata K, Tajima K, Nakamura M, Nagamine T, Aminov RI, Benno Y. Phenotypic characterization of polysaccharidases produced by four Prevotella type strains. Curr Microbiol. 2000;41(1):45–9. 10.1007/s002840010089 10919398

[27] Pitta DW, Pinchak WE, Dowd SE, Osterstock J, Gontcharova V, Youn E (2010). Rumen bacterial diversity dynamics associated with changing from bermudagrass hay to grazed winter wheat diets. Microl Ecol.

[28] Krause DO, Denman SE, Mackie RI, Morrison M, Rae AL, Attwood GT, McSweeney CS. Opportunities to improve fiber degradation in the rumen: microbiology, ecology, and genomics. FEMS Microbiol Immunol. 2003;27(5):663–93. 10.1016/S0168-6445(03)00072-X 14638418

[29] Danscher AM, Li S, Andersen PH, Khafipour E, Kristensen NB, Plaizier JC (2015). Indicators of induced subacute ruminal acidosis (SARA) in Danish Holstein cows. Acta Vet Scand.

[30] AOAC International (2003). Official Methods of Analysis.

[31] Van Soest PJ, Robertson JB, Lewis BA (1991). Methods for dietary fiber, neutral detergent fiber, and non-starch polysaccharides in relation to animal nutrition. J Dairy Sci.

[32] AOAC International (2005). Official Methods of Analysis.

[33] Caporaso JG, Lauber CL, Walters WA, Berg-Lyons D, Huntley J, Fierer N (2012). Ultra-high-throughput microbial community analysis on the Illumina HiSeq and MiSeq platforms. ISME J.

[34] Wang RF, Cao WW, Cerniglia CE (1997). PCR detection of Ruminococcus spp. in human and animal faecal samples. Mol Cell Probes.

[35] Ozutsumi Y, Tajima K, Takenaka A, Itabashi H (2006). Real-time PCR detection of the effects of protozoa on rumen bacteria in cattle. Curr Microbiol.

[36] Denman SE, McSweeney CS (2006). Development of a real-time PCR assay for monitoring anaerobic fungal and cellulolytic bacterial populations within the rumen. FEMS Microbiol Ecol.

[37] Masella A, Bartram A, Truszkowski J, Brown D, Neufeld J (2012). PANDAseq: paired-end assembler for illumina sequences. BMC Bioinformatics.

[38] Caporaso JG, Bittinger K, Bushman FD, DeSantis TZ, Andersen GL, Knight R (2010). PyNAST: a flexible tool for aligning sequences to a template alignment. Bioinformatics.

[39] Edgar RC, Haas BJ, Clemente JC, Quince C, Knight R (2011). UCHIME improves sensitivity and speed of chimera detection. Bioinformatics.

[40] Edgar RC (2010). Search and clustering orders of magnitude faster than BLAST. Bioinformatics.

[41] Wang Q, Garrity GM, Tiedje JM, Cole JR (2007). Naive Bayesian classifier for rapid assignment of rRNA sequences into the new bacterial taxonomy. Appl Environ Microbiol.

[42] Anderson MJ (2005). Permutational multivariate analysis of variance.

[43] Lozupone C, Hamady M, Knight R (2006). UniFrac--an online tool for comparing microbial community diversity in a phylogenetic context. BMC Bioinformatics.

[44] Love MI, Wolfgang H, Anders S. Moderated estimation of fold change and dispersion for RNA-Seq data with DESeq2. bioRxiv preprint; 2014. 10.1186/s13059-014-0550-8 PMC4302049 25516281

